# Bis{(*E*)-*N*′-[2,4-bis(trifluoro­meth­yl)benzyl­idene]isonicotinohydrazide} monohydrate

**DOI:** 10.1107/S1600536810025493

**Published:** 2010-07-03

**Authors:** H. S. Naveenkumar, Amirin Sadikun, Pazilah Ibrahim, Chin Sing Yeap, Hoong-Kun Fun

**Affiliations:** a School of Pharmaceutical Sciences, Universiti Sains Malaysia, 11800 USM, Penang, Malaysia; b X-ray Crystallography Unit, School of Physics, Universiti Sains Malaysia, 11800 USM, Penang, Malaysia

## Abstract

The asymmetric unit of the title compound, 2C_15_H_9_F_6_N_3_O·H_2_O, contains two independent Schiff base mol­ecules and one water mol­ecule. Both Schiff base mol­ecules exist in an *E* configuration with respect to the C=N double bonds and the dihedral angles between the benzene and the pyridine rings in the two mol­ecules are 17.53 (12) and 20.62 (12)°. In the crystal structure, mol­ecules are linked by inter­molecular N—H⋯O and C—H⋯O hydrogen bonds into infinite one-dimensional chains along the *a* axis. In addition, inter­molecular O—H⋯N, O—H⋯F, C—H⋯F and C—H⋯O hydrogen bonds further link these chains into a three-dimensional network. Weak π–π inter­actions with centroid–centroid distances in the range 3.6495 (17)–3.7092 (16) Å are also observed.

## Related literature

For applications of isoniazid derivatives, see: Janin (2007 ▶); Maccari *et al.* (2005 ▶); Slayden & Barry (2000 ▶); Kahwa *et al.* (1986 ▶). For the preparation of the title compound, see: Lourenco *et al.* (2008 ▶). For related structures, see: Naveenkumar *et al.* (2009 ▶, 2010*a* ▶,*b* ▶). For the stability of the temperature controller used for the data collection, see: Cosier & Glazer (1986 ▶).
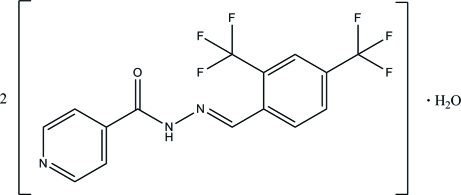


## Experimental

### 

#### Crystal data

2C_15_H_9_F_6_N_3_O·H_2_O
*M* *_r_* = 740.52
Monoclinic, 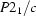

*a* = 8.2487 (18) Å
*b* = 26.649 (6) Å
*c* = 14.779 (3) Å
β = 109.076 (10)°
*V* = 3070.3 (11) Å^3^
*Z* = 4
Mo *K*α radiation
μ = 0.16 mm^−1^
*T* = 100 K
0.59 × 0.17 × 0.13 mm

#### Data collection

Bruker APEXII DUO CCD area-detector diffractometer
Absorption correction: multi-scan (*SADABS*; Bruker, 2009 ▶) *T*_min_ = 0.914, *T*_max_ = 0.980
29846 measured reflections
7030 independent reflections
5239 reflections with *I* > 2σ(*I*)
*R*_int_ = 0.030

#### Refinement

*R*[*F* ^2^ > 2σ(*F* ^2^)] = 0.057
*wR*(*F* ^2^) = 0.162
*S* = 1.02
7030 reflections
460 parameters
H-atom parameters constrained
Δρ_max_ = 0.67 e Å^−3^
Δρ_min_ = −0.68 e Å^−3^

### 

Data collection: *APEX2* (Bruker, 2009 ▶); cell refinement: *SAINT* (Bruker, 2009 ▶); data reduction: *SAINT*; program(s) used to solve structure: *SHELXTL* (Sheldrick, 2008 ▶); program(s) used to refine structure: *SHELXTL*; molecular graphics: *SHELXTL*; software used to prepare material for publication: *SHELXTL* and *PLATON* (Spek, 2009 ▶).

## Supplementary Material

Crystal structure: contains datablocks global, I. DOI: 10.1107/S1600536810025493/lh5074sup1.cif

Structure factors: contains datablocks I. DOI: 10.1107/S1600536810025493/lh5074Isup2.hkl

Additional supplementary materials:  crystallographic information; 3D view; checkCIF report

## Figures and Tables

**Table 1:** Hydrogen-bond geometry (Å, °)

*D*—H⋯*A*	*D*—H	H⋯*A*	*D*⋯*A*	*D*—H⋯*A*
N2*A*—H2*NA*⋯O1*B*	0.86	2.05	2.856 (3)	156
N2*B*—H2*NB*⋯O1*A*^i^	0.86	2.10	2.908 (3)	155
C7*A*—H7*A*⋯O1*B*	0.93	2.23	3.055 (3)	147
C7*B*—H7*B*⋯O1*A*^i^	0.93	2.36	3.158 (3)	144
C2*B*—H2*B*⋯F1*A*^ii^	0.93	2.52	3.294 (3)	141
C9*A*—H9*A*⋯F2*B*^iii^	0.93	2.41	3.162 (4)	138
C12*B*—H12*B*⋯O1*W*^iv^	0.93	2.58	3.408 (5)	149
O1*W*—H1*WA*⋯F2*B*^iv^	0.84	2.01	2.845 (5)	180
O1*W*—H1*WB*⋯N1*B*^v^	0.84	2.09	2.932 (5)	180

Symmetry codes: (i) 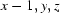; (ii) 
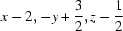
; (iii) 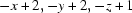; (iv) 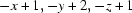; (v) 
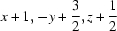
.

**Fig. 1.**
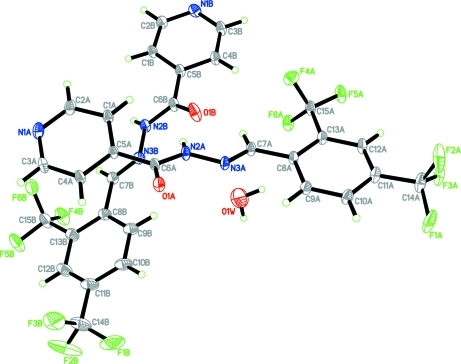
The asymmetric unit of the title compound with atom labels and 30% probability ellipsoids for non-H atoms.

**Fig. 2.**
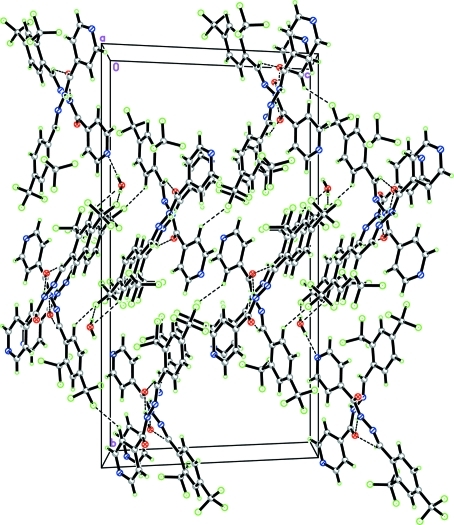
The crystal packing of title compound, viewed along the a axis, showing the molecules are linked into a 3-D network. Intermolecular hydrogen bonds are shown as dashed lines.

## supplementary crystallographic information

### Comment

In the search of new compounds, isoniazid derivatives have been found to possess potential tuberculostatic activity (Janin, 2007; Maccari *et al.*, 2005; Slayden & Barry, 2000). As a part of a current work of synthesis of such derivatives, in this paper we present the crystal structure of the title compound.

The asymmetric unit consists of two Schiff base molecules [A and B] and one water molecule (Fig. 1). The geometric parameters are comparable to those related structures (Naveenkumar *et al.*, 2009, 2010*a*, *b*). The molecules exist in *E* configurations with respect to the C7A═N3A and C7B═N3B double bonds. The dihedral angles between the benzene ring and the pyridine ring in molecules *A* and *B* are 17.53 (12) and 20.62 (12)°, respectively.

In the crystal structure, the molecules are linked by intermolecular N2A—H2NA···O1B, C7A—H7A···O1B, N2B—H2NB···O1A and C7B—H7B···O1A hydrogen bonds (Table 1) into infinite one-dimensional chains along the *a* axis. Intermolecular O1W—H1WB···N1B, O1W—H1WA···F2B, C9A—H9A···F2B, C2B—H2B···F1A and C12B—H12B···O1W hydrogen bonds further link these chains into a three-dimensional network (Fig. 2, Table 1). Weak π–π interactions are also observed with *Cg*1···*Cg*1^vi^ = 3.6529 (17) Å, *Cg*2···*Cg*3^v^ = 3.7092 (16) Å and *Cg*4···*Cg*4^iv^ = 3.6495 (17) Å [*Cg*1, *Cg*2, *Cg*3 and *Cg*4 are centroids of C1A/C2A/N1A/C3A/C4A/C5A, C8A–C13A, C1B/C2B/N1B/C3B/C4B/C5B and C8B–C13B rings, respectively; symmetry code: (iv) 1 - *x*,2 - *y*,1 - *z*; (v) 1 + *x*,3/2 - *y*,1/2 + *z*; (vi) 1 - *x*,2 - *y*,-*z*].

### Experimental

The isoniazid derivative was prepared following the procedure by Lourenco *et al*., 2008. The title compound was prepared by reaction between the 2,4-bis(trifluoro-methyl)benzaldehyde (1.0 eq) with isoniazid (1.0 eq) in ethanol/water. After stirring for 1–3 h at room temperature, the resulting mixture was concentrated under reduced pressure. The residue, purified by washing with cold ethanol and diethyl ether, afforded the pure derivative. The colourless single-crystals suitable for X-ray analysis was obtained by recrystalization from ethanol.

### Refinement

Hydrogen atoms were positioned geometrically [N-H = 0.86Å, O-H = 0.84Å and C–H = 0.93 Å] and refined using a riding model, with *U*_iso_(H) = 1.2*U*_eq_(C,N) and 1.5*U*_eq_(O). The H atoms of the water molecule were included in positions which give ideal geometry for hydrogen bonds.

### Crystal data

**Table:**

2C_15_H_9_F_6_N_3_O·H_2_O	*F*(000) = 1496
*M**_r_* = 740.52	*D*_x_ = 1.602 Mg m^−^^3^
Monoclinic, *P*2_1_/*c*	Mo *K*α radiation, λ = 0.71073 Å
Hall symbol: -P 2ybc	Cell parameters from 8714 reflections
*a* = 8.2487 (18) Å	θ = 2.7–30.0°
*b* = 26.649 (6) Å	µ = 0.16 mm^−^^1^
*c* = 14.779 (3) Å	*T* = 100 K
β = 109.076 (10)°	Needle, colourless
*V* = 3070.3 (11) Å^3^	0.59 × 0.17 × 0.13 mm
*Z* = 4	

### Data collection

**Table:**

Bruker APEXII DUO CCD area-detector diffractometer	7030 independent reflections
Radiation source: fine-focus sealed tube	5239 reflections with *I* > 2σ(*I*)
graphite	*R*_int_ = 0.030
φ and ω scans	θ_max_ = 27.5°, θ_min_ = 1.5°
Absorption correction: multi-scan (*SADABS*; Bruker, 2009)	*h* = −10→10
*T*_min_ = 0.914, *T*_max_ = 0.980	*k* = −34→34
29846 measured reflections	*l* = −19→19

### Refinement

**Table:**

Refinement on *F*^2^	Primary atom site location: structure-invariant direct methods
Least-squares matrix: full	Secondary atom site location: difference Fourier map
*R*[*F*^2^ > 2σ(*F*^2^)] = 0.057	Hydrogen site location: inferred from neighbouring sites
*wR*(*F*^2^) = 0.162	H-atom parameters constrained
*S* = 1.02	*w* = 1/[σ^2^(*F*_o_^2^) + (0.0688*P*)^2^ + 3.4968*P*] where *P* = (*F*_o_^2^ + 2*F*_c_^2^)/3
7030 reflections	(Δ/σ)_max_ = 0.001
460 parameters	Δρ_max_ = 0.67 e Å^−^^3^
0 restraints	Δρ_min_ = −0.68 e Å^−^^3^

### Special details

**Table:**

Experimental. The crystal was placed in the cold stream of an Oxford Cryosystems Cobra open-flow nitrogen cryostat (Cosier & Glazer, 1986) operating at 100.0 (1) K.
Geometry. All e.s.d.'s (except the e.s.d. in the dihedral angle between two l.s. planes) are estimated using the full covariance matrix. The cell e.s.d.'s are taken into account individually in the estimation of e.s.d.'s in distances, angles and torsion angles; correlations between e.s.d.'s in cell parameters are only used when they are defined by crystal symmetry. An approximate (isotropic) treatment of cell e.s.d.'s is used for estimating e.s.d.'s involving l.s. planes.
Refinement. Refinement of *F*^2^ against ALL reflections. The weighted *R*-factor *wR* and goodness of fit *S* are based on *F*^2^, conventional *R*-factors *R* are based on *F*, with *F* set to zero for negative *F*^2^. The threshold expression of *F*^2^ > σ(*F*^2^) is used only for calculating *R*-factors(gt) *etc*. and is not relevant to the choice of reflections for refinement. *R*-factors based on *F*^2^ are statistically about twice as large as those based on *F*, and *R*- factors based on ALL data will be even larger.

### Fractional atomic coordinates and isotropic or equivalent isotropic displacement parameters (Å^2^)

**Table:**

	*x*	*y*	*z*	*U*_iso_*/*U*_eq_	
F1A	1.3386 (2)	0.67680 (7)	0.46754 (17)	0.0730 (6)	
F2A	1.2638 (3)	0.64562 (7)	0.32715 (17)	0.0769 (7)	
F3A	1.1394 (2)	0.62210 (6)	0.42543 (13)	0.0516 (4)	
F4A	0.53442 (19)	0.72504 (6)	0.14372 (11)	0.0436 (4)	
F5A	0.57719 (19)	0.66261 (5)	0.24013 (13)	0.0453 (4)	
F6A	0.49749 (17)	0.73324 (5)	0.27950 (11)	0.0372 (3)	
O1A	0.83293 (19)	0.95521 (6)	0.18897 (12)	0.0307 (4)	
N1A	0.2710 (3)	1.04566 (8)	0.05493 (16)	0.0387 (5)	
N2A	0.6377 (2)	0.89622 (6)	0.19410 (14)	0.0276 (4)	
H2NA	0.5315	0.8888	0.1828	0.033*	
N3A	0.7647 (2)	0.86131 (6)	0.23267 (14)	0.0266 (4)	
C1A	0.3807 (3)	0.96185 (8)	0.07092 (17)	0.0285 (5)	
H1A	0.3614	0.9281	0.0552	0.034*	
C2A	0.2523 (3)	0.99707 (9)	0.03320 (18)	0.0346 (5)	
H2A	0.1481	0.9862	−0.0093	0.041*	
C3A	0.4233 (3)	1.06017 (8)	0.11472 (19)	0.0366 (5)	
H3A	0.4379	1.0939	0.1312	0.044*	
C4A	0.5604 (3)	1.02840 (8)	0.15360 (17)	0.0298 (5)	
H4A	0.6652	1.0407	0.1930	0.036*	
C5A	0.5379 (3)	0.97783 (7)	0.13241 (15)	0.0237 (4)	
C6A	0.6844 (3)	0.94253 (7)	0.17445 (16)	0.0246 (4)	
C7A	0.7099 (3)	0.81749 (8)	0.24256 (17)	0.0286 (5)	
H7A	0.5928	0.8111	0.2241	0.034*	
C8A	0.8339 (3)	0.77756 (8)	0.28328 (16)	0.0265 (4)	
C9A	1.0062 (3)	0.78936 (8)	0.32681 (17)	0.0292 (5)	
H9A	1.0408	0.8227	0.3305	0.035*	
C10A	1.1269 (3)	0.75252 (9)	0.36459 (17)	0.0330 (5)	
H10A	1.2418	0.7609	0.3934	0.040*	
C11A	1.0749 (3)	0.70289 (9)	0.35908 (18)	0.0339 (5)	
C12A	0.9041 (3)	0.69018 (8)	0.31789 (18)	0.0328 (5)	
H12A	0.8702	0.6568	0.3158	0.039*	
C13A	0.7835 (3)	0.72720 (8)	0.27974 (17)	0.0279 (5)	
C14A	1.2037 (3)	0.66203 (10)	0.3951 (2)	0.0460 (7)	
C15A	0.5987 (3)	0.71217 (8)	0.23552 (18)	0.0324 (5)	
F1B	0.9350 (3)	1.07132 (10)	0.53923 (16)	0.0896 (8)	
F2B	0.7582 (4)	1.12296 (12)	0.5572 (2)	0.1222 (12)	
F3B	0.8325 (3)	1.12608 (8)	0.43264 (16)	0.0815 (7)	
F4B	0.0666 (2)	1.04165 (6)	0.38057 (14)	0.0563 (5)	
F5B	0.1590 (3)	1.11618 (6)	0.37229 (16)	0.0672 (6)	
F6B	0.0833 (2)	1.06925 (6)	0.24796 (14)	0.0566 (5)	
O1B	0.3278 (2)	0.84289 (7)	0.17464 (18)	0.0525 (6)	
N1B	−0.2255 (3)	0.75716 (8)	0.00655 (16)	0.0357 (5)	
N2B	0.1387 (2)	0.89800 (7)	0.19927 (14)	0.0291 (4)	
H2NB	0.0338	0.9061	0.1909	0.035*	
N3B	0.2726 (2)	0.92809 (7)	0.25030 (14)	0.0309 (4)	
C1B	−0.1252 (3)	0.84074 (8)	0.05378 (17)	0.0298 (5)	
H1B	−0.1484	0.8750	0.0505	0.036*	
C2B	−0.2489 (3)	0.80662 (9)	0.00503 (18)	0.0337 (5)	
H2B	−0.3558	0.8190	−0.0313	0.040*	
C3B	−0.0697 (3)	0.74026 (9)	0.05774 (19)	0.0360 (5)	
H3B	−0.0498	0.7059	0.0594	0.043*	
C4B	0.0630 (3)	0.77130 (8)	0.10816 (19)	0.0335 (5)	
H4B	0.1699	0.7580	0.1420	0.040*	
C5B	0.0346 (3)	0.82255 (8)	0.10771 (17)	0.0280 (5)	
C6B	0.1793 (3)	0.85540 (8)	0.16307 (19)	0.0326 (5)	
C7B	0.2332 (3)	0.96770 (9)	0.28583 (17)	0.0311 (5)	
H7B	0.1193	0.9757	0.2769	0.037*	
C8B	0.3734 (3)	1.00068 (9)	0.34169 (17)	0.0322 (5)	
C9B	0.5420 (3)	0.98353 (10)	0.36821 (17)	0.0359 (5)	
H9B	0.5634	0.9509	0.3530	0.043*	
C10B	0.6783 (3)	1.01409 (11)	0.41673 (18)	0.0418 (6)	
H10B	0.7904	1.0024	0.4325	0.050*	
C11B	0.6459 (4)	1.06192 (11)	0.44127 (18)	0.0448 (7)	
C12B	0.4801 (4)	1.07976 (10)	0.41867 (19)	0.0434 (6)	
H12B	0.4600	1.1119	0.4370	0.052*	
C13B	0.3433 (3)	1.04929 (9)	0.36825 (18)	0.0372 (6)	
C14B	0.7939 (5)	1.09561 (14)	0.4928 (2)	0.0605 (9)	
C15B	0.1644 (4)	1.06914 (10)	0.3428 (2)	0.0480 (7)	
O1W	0.5352 (5)	0.82564 (14)	0.4292 (3)	0.1324 (16)	
H1WA	0.4487	0.8409	0.4331	0.199*	
H1WB	0.6041	0.8020	0.4512	0.199*	

### Atomic displacement parameters (Å^2^)

**Table:**

	*U*^11^	*U*^22^	*U*^33^	*U*^12^	*U*^13^	*U*^23^
F1A	0.0290 (9)	0.0562 (11)	0.1136 (17)	0.0121 (8)	−0.0043 (9)	0.0302 (11)
F2A	0.0817 (15)	0.0613 (12)	0.1128 (17)	0.0463 (11)	0.0660 (14)	0.0371 (11)
F3A	0.0366 (9)	0.0357 (8)	0.0809 (12)	0.0138 (7)	0.0172 (8)	0.0275 (8)
F4A	0.0327 (8)	0.0454 (8)	0.0458 (8)	−0.0101 (6)	0.0034 (6)	0.0051 (7)
F5A	0.0328 (8)	0.0190 (6)	0.0825 (11)	−0.0040 (6)	0.0165 (8)	0.0044 (7)
F6A	0.0224 (7)	0.0308 (7)	0.0593 (9)	0.0017 (5)	0.0146 (6)	0.0057 (6)
O1A	0.0182 (8)	0.0218 (7)	0.0493 (10)	−0.0021 (6)	0.0074 (7)	0.0024 (7)
N1A	0.0335 (11)	0.0314 (10)	0.0519 (13)	0.0132 (9)	0.0148 (10)	0.0085 (9)
N2A	0.0159 (8)	0.0177 (8)	0.0468 (11)	0.0019 (6)	0.0071 (8)	0.0052 (7)
N3A	0.0191 (9)	0.0193 (8)	0.0388 (10)	0.0037 (7)	0.0061 (7)	0.0040 (7)
C1A	0.0211 (10)	0.0203 (10)	0.0425 (12)	−0.0008 (8)	0.0083 (9)	0.0023 (9)
C2A	0.0223 (11)	0.0314 (12)	0.0477 (14)	0.0049 (9)	0.0083 (10)	0.0068 (10)
C3A	0.0455 (15)	0.0180 (10)	0.0490 (14)	0.0064 (10)	0.0191 (12)	0.0015 (9)
C4A	0.0308 (12)	0.0186 (10)	0.0400 (12)	−0.0023 (8)	0.0115 (10)	−0.0004 (9)
C5A	0.0182 (10)	0.0167 (9)	0.0370 (11)	−0.0001 (7)	0.0099 (8)	0.0028 (8)
C6A	0.0189 (10)	0.0170 (9)	0.0362 (11)	−0.0005 (7)	0.0069 (8)	−0.0004 (8)
C7A	0.0179 (10)	0.0207 (10)	0.0447 (12)	0.0010 (8)	0.0068 (9)	0.0049 (9)
C8A	0.0216 (10)	0.0208 (10)	0.0358 (11)	0.0020 (8)	0.0077 (9)	0.0046 (8)
C9A	0.0231 (11)	0.0235 (10)	0.0389 (12)	−0.0009 (8)	0.0072 (9)	0.0040 (9)
C10A	0.0212 (11)	0.0335 (12)	0.0415 (13)	0.0030 (9)	0.0065 (9)	0.0081 (10)
C11A	0.0275 (12)	0.0298 (11)	0.0458 (13)	0.0087 (9)	0.0137 (10)	0.0113 (10)
C12A	0.0277 (12)	0.0217 (10)	0.0502 (14)	0.0053 (9)	0.0147 (10)	0.0079 (9)
C13A	0.0217 (11)	0.0219 (10)	0.0400 (12)	0.0022 (8)	0.0101 (9)	0.0049 (9)
C14A	0.0290 (13)	0.0408 (14)	0.0705 (19)	0.0131 (11)	0.0193 (13)	0.0205 (13)
C15A	0.0267 (11)	0.0197 (10)	0.0501 (14)	−0.0001 (8)	0.0116 (10)	0.0053 (9)
F1B	0.0616 (14)	0.1076 (18)	0.0721 (14)	−0.0456 (13)	−0.0158 (11)	0.0014 (12)
F2B	0.109 (2)	0.157 (3)	0.117 (2)	−0.089 (2)	0.0587 (17)	−0.099 (2)
F3B	0.0739 (14)	0.0757 (14)	0.0837 (14)	−0.0487 (12)	0.0104 (11)	0.0088 (11)
F4B	0.0472 (10)	0.0400 (9)	0.0904 (13)	−0.0042 (7)	0.0346 (9)	−0.0085 (8)
F5B	0.0679 (13)	0.0311 (8)	0.1054 (16)	−0.0032 (8)	0.0319 (11)	−0.0196 (9)
F6B	0.0495 (10)	0.0355 (8)	0.0743 (12)	0.0027 (7)	0.0057 (9)	0.0018 (8)
O1B	0.0177 (9)	0.0292 (9)	0.1087 (17)	−0.0001 (7)	0.0179 (9)	−0.0146 (10)
N1B	0.0288 (10)	0.0309 (10)	0.0492 (12)	−0.0074 (8)	0.0151 (9)	−0.0048 (9)
N2B	0.0140 (8)	0.0239 (9)	0.0475 (11)	−0.0008 (7)	0.0072 (8)	−0.0026 (8)
N3B	0.0197 (9)	0.0273 (9)	0.0421 (11)	−0.0050 (7)	0.0054 (8)	−0.0004 (8)
C1B	0.0224 (11)	0.0239 (10)	0.0436 (13)	0.0035 (8)	0.0113 (9)	−0.0010 (9)
C2B	0.0213 (11)	0.0340 (12)	0.0441 (13)	0.0002 (9)	0.0083 (9)	−0.0028 (10)
C3B	0.0341 (13)	0.0216 (11)	0.0552 (15)	−0.0009 (9)	0.0185 (11)	−0.0001 (10)
C4B	0.0250 (11)	0.0232 (11)	0.0517 (14)	0.0024 (9)	0.0116 (10)	0.0016 (10)
C5B	0.0199 (10)	0.0219 (10)	0.0446 (13)	−0.0003 (8)	0.0135 (9)	−0.0011 (9)
C6B	0.0188 (11)	0.0226 (10)	0.0548 (14)	0.0003 (8)	0.0098 (10)	0.0011 (10)
C7B	0.0221 (11)	0.0294 (11)	0.0411 (12)	−0.0028 (9)	0.0094 (9)	−0.0010 (9)
C8B	0.0294 (12)	0.0321 (12)	0.0349 (12)	−0.0089 (9)	0.0100 (9)	−0.0013 (9)
C9B	0.0284 (12)	0.0429 (13)	0.0355 (12)	−0.0084 (10)	0.0091 (10)	−0.0034 (10)
C10B	0.0317 (13)	0.0570 (17)	0.0349 (13)	−0.0158 (12)	0.0085 (10)	−0.0050 (11)
C11B	0.0447 (16)	0.0560 (17)	0.0330 (12)	−0.0245 (13)	0.0118 (11)	−0.0063 (11)
C12B	0.0554 (17)	0.0361 (13)	0.0421 (14)	−0.0186 (12)	0.0206 (12)	−0.0072 (11)
C13B	0.0402 (14)	0.0325 (12)	0.0407 (13)	−0.0098 (10)	0.0157 (11)	−0.0028 (10)
C14B	0.062 (2)	0.066 (2)	0.0557 (18)	−0.0345 (17)	0.0222 (17)	−0.0206 (16)
C15B	0.0508 (17)	0.0283 (12)	0.0659 (19)	−0.0044 (11)	0.0207 (14)	−0.0073 (12)
O1W	0.119 (3)	0.109 (3)	0.134 (3)	0.069 (2)	−0.006 (2)	−0.044 (2)

### Geometric parameters (Å, °)

**Table:**

F1A—C14A	1.326 (4)	F2B—C14B	1.306 (4)
F2A—C14A	1.331 (4)	F3B—C14B	1.317 (4)
F3A—C14A	1.331 (3)	F4B—C15B	1.339 (3)
F4A—C15A	1.330 (3)	F5B—C15B	1.333 (3)
F5A—C15A	1.337 (3)	F6B—C15B	1.341 (4)
F6A—C15A	1.338 (3)	O1B—C6B	1.226 (3)
O1A—C6A	1.221 (3)	N1B—C2B	1.331 (3)
N1A—C2A	1.331 (3)	N1B—C3B	1.339 (3)
N1A—C3A	1.336 (3)	N2B—C6B	1.343 (3)
N2A—C6A	1.352 (3)	N2B—N3B	1.374 (3)
N2A—N3A	1.377 (2)	N2B—H2NB	0.8600
N2A—H2NA	0.8600	N3B—C7B	1.268 (3)
N3A—C7A	1.277 (3)	C1B—C2B	1.381 (3)
C1A—C5A	1.385 (3)	C1B—C5B	1.386 (3)
C1A—C2A	1.388 (3)	C1B—H1B	0.9300
C1A—H1A	0.9300	C2B—H2B	0.9300
C2A—H2A	0.9300	C3B—C4B	1.380 (3)
C3A—C4A	1.379 (3)	C3B—H3B	0.9300
C3A—H3A	0.9300	C4B—C5B	1.385 (3)
C4A—C5A	1.382 (3)	C4B—H4B	0.9300
C4A—H4A	0.9300	C5B—C6B	1.490 (3)
C5A—C6A	1.496 (3)	C7B—C8B	1.472 (3)
C7A—C8A	1.462 (3)	C7B—H7B	0.9300
C7A—H7A	0.9300	C8B—C9B	1.393 (4)
C8A—C9A	1.392 (3)	C8B—C13B	1.399 (3)
C8A—C13A	1.401 (3)	C9B—C10B	1.383 (3)
C9A—C10A	1.380 (3)	C9B—H9B	0.9300
C9A—H9A	0.9300	C10B—C11B	1.375 (4)
C10A—C11A	1.385 (3)	C10B—H10B	0.9300
C10A—H10A	0.9300	C11B—C12B	1.382 (4)
C11A—C12A	1.382 (3)	C11B—C14B	1.506 (4)
C11A—C14A	1.493 (3)	C12B—C13B	1.392 (4)
C12A—C13A	1.384 (3)	C12B—H12B	0.9300
C12A—H12A	0.9300	C13B—C15B	1.496 (4)
C13A—C15A	1.503 (3)	O1W—H1WA	0.8400
F1B—C14B	1.313 (5)	O1W—H1WB	0.8400
			
C2A—N1A—C3A	116.8 (2)	C6B—N2B—N3B	116.85 (18)
C6A—N2A—N3A	118.29 (17)	C6B—N2B—H2NB	121.6
C6A—N2A—H2NA	120.9	N3B—N2B—H2NB	121.6
N3A—N2A—H2NA	120.9	C7B—N3B—N2B	116.41 (19)
C7A—N3A—N2A	114.54 (18)	C2B—C1B—C5B	118.1 (2)
C5A—C1A—C2A	118.9 (2)	C2B—C1B—H1B	120.9
C5A—C1A—H1A	120.5	C5B—C1B—H1B	120.9
C2A—C1A—H1A	120.5	N1B—C2B—C1B	124.4 (2)
N1A—C2A—C1A	123.2 (2)	N1B—C2B—H2B	117.8
N1A—C2A—H2A	118.4	C1B—C2B—H2B	117.8
C1A—C2A—H2A	118.4	N1B—C3B—C4B	123.3 (2)
N1A—C3A—C4A	124.3 (2)	N1B—C3B—H3B	118.3
N1A—C3A—H3A	117.8	C4B—C3B—H3B	118.3
C4A—C3A—H3A	117.8	C3B—C4B—C5B	119.0 (2)
C3A—C4A—C5A	118.3 (2)	C3B—C4B—H4B	120.5
C3A—C4A—H4A	120.9	C5B—C4B—H4B	120.5
C5A—C4A—H4A	120.9	C4B—C5B—C1B	118.4 (2)
C4A—C5A—C1A	118.4 (2)	C4B—C5B—C6B	118.2 (2)
C4A—C5A—C6A	119.25 (19)	C1B—C5B—C6B	123.4 (2)
C1A—C5A—C6A	122.32 (18)	O1B—C6B—N2B	122.8 (2)
O1A—C6A—N2A	123.70 (19)	O1B—C6B—C5B	120.0 (2)
O1A—C6A—C5A	121.77 (18)	N2B—C6B—C5B	117.14 (19)
N2A—C6A—C5A	114.53 (18)	N3B—C7B—C8B	118.0 (2)
N3A—C7A—C8A	119.11 (19)	N3B—C7B—H7B	121.0
N3A—C7A—H7A	120.4	C8B—C7B—H7B	121.0
C8A—C7A—H7A	120.4	C9B—C8B—C13B	118.5 (2)
C9A—C8A—C13A	118.67 (19)	C9B—C8B—C7B	119.4 (2)
C9A—C8A—C7A	119.81 (19)	C13B—C8B—C7B	122.1 (2)
C13A—C8A—C7A	121.51 (19)	C10B—C9B—C8B	121.3 (3)
C10A—C9A—C8A	121.3 (2)	C10B—C9B—H9B	119.3
C10A—C9A—H9A	119.4	C8B—C9B—H9B	119.3
C8A—C9A—H9A	119.4	C11B—C10B—C9B	119.2 (3)
C9A—C10A—C11A	119.2 (2)	C11B—C10B—H10B	120.4
C9A—C10A—H10A	120.4	C9B—C10B—H10B	120.4
C11A—C10A—H10A	120.4	C10B—C11B—C12B	121.1 (2)
C12A—C11A—C10A	120.8 (2)	C10B—C11B—C14B	119.4 (3)
C12A—C11A—C14A	118.9 (2)	C12B—C11B—C14B	119.4 (3)
C10A—C11A—C14A	120.3 (2)	C11B—C12B—C13B	119.6 (3)
C11A—C12A—C13A	119.9 (2)	C11B—C12B—H12B	120.2
C11A—C12A—H12A	120.1	C13B—C12B—H12B	120.2
C13A—C12A—H12A	120.1	C12B—C13B—C8B	120.2 (3)
C12A—C13A—C8A	120.2 (2)	C12B—C13B—C15B	119.3 (2)
C12A—C13A—C15A	118.6 (2)	C8B—C13B—C15B	120.5 (2)
C8A—C13A—C15A	121.20 (19)	F2B—C14B—F1B	105.4 (3)
F1A—C14A—F3A	106.7 (2)	F2B—C14B—F3B	108.0 (3)
F1A—C14A—F2A	106.6 (2)	F1B—C14B—F3B	106.8 (3)
F3A—C14A—F2A	106.2 (2)	F2B—C14B—C11B	111.3 (3)
F1A—C14A—C11A	112.5 (2)	F1B—C14B—C11B	113.8 (3)
F3A—C14A—C11A	112.8 (2)	F3B—C14B—C11B	111.2 (3)
F2A—C14A—C11A	111.6 (2)	F5B—C15B—F4B	106.8 (2)
F4A—C15A—F5A	106.9 (2)	F5B—C15B—F6B	106.3 (2)
F4A—C15A—F6A	106.45 (19)	F4B—C15B—F6B	105.9 (2)
F5A—C15A—F6A	105.99 (19)	F5B—C15B—C13B	112.7 (2)
F4A—C15A—C13A	112.60 (19)	F4B—C15B—C13B	112.4 (2)
F5A—C15A—C13A	111.95 (19)	F6B—C15B—C13B	112.2 (2)
F6A—C15A—C13A	112.5 (2)	H1WA—O1W—H1WB	145.0
C2B—N1B—C3B	116.7 (2)		
			
C6A—N2A—N3A—C7A	−175.0 (2)	C6B—N2B—N3B—C7B	−178.6 (2)
C3A—N1A—C2A—C1A	1.3 (4)	C3B—N1B—C2B—C1B	−1.3 (4)
C5A—C1A—C2A—N1A	−1.7 (4)	C5B—C1B—C2B—N1B	0.3 (4)
C2A—N1A—C3A—C4A	0.8 (4)	C2B—N1B—C3B—C4B	0.6 (4)
N1A—C3A—C4A—C5A	−2.4 (4)	N1B—C3B—C4B—C5B	1.1 (4)
C3A—C4A—C5A—C1A	2.0 (3)	C3B—C4B—C5B—C1B	−2.1 (4)
C3A—C4A—C5A—C6A	−179.5 (2)	C3B—C4B—C5B—C6B	179.6 (2)
C2A—C1A—C5A—C4A	−0.1 (3)	C2B—C1B—C5B—C4B	1.4 (4)
C2A—C1A—C5A—C6A	−178.6 (2)	C2B—C1B—C5B—C6B	179.6 (2)
N3A—N2A—C6A—O1A	0.0 (3)	N3B—N2B—C6B—O1B	0.6 (4)
N3A—N2A—C6A—C5A	179.77 (18)	N3B—N2B—C6B—C5B	179.9 (2)
C4A—C5A—C6A—O1A	−33.3 (3)	C4B—C5B—C6B—O1B	29.3 (4)
C1A—C5A—C6A—O1A	145.2 (2)	C1B—C5B—C6B—O1B	−148.9 (3)
C4A—C5A—C6A—N2A	146.9 (2)	C4B—C5B—C6B—N2B	−150.0 (2)
C1A—C5A—C6A—N2A	−34.6 (3)	C1B—C5B—C6B—N2B	31.8 (4)
N2A—N3A—C7A—C8A	−179.7 (2)	N2B—N3B—C7B—C8B	179.5 (2)
N3A—C7A—C8A—C9A	12.0 (3)	N3B—C7B—C8B—C9B	−11.4 (3)
N3A—C7A—C8A—C13A	−168.1 (2)	N3B—C7B—C8B—C13B	167.7 (2)
C13A—C8A—C9A—C10A	1.1 (4)	C13B—C8B—C9B—C10B	−2.3 (4)
C7A—C8A—C9A—C10A	−179.0 (2)	C7B—C8B—C9B—C10B	176.9 (2)
C8A—C9A—C10A—C11A	−0.1 (4)	C8B—C9B—C10B—C11B	1.7 (4)
C9A—C10A—C11A—C12A	−1.1 (4)	C9B—C10B—C11B—C12B	0.3 (4)
C9A—C10A—C11A—C14A	177.1 (2)	C9B—C10B—C11B—C14B	−179.1 (3)
C10A—C11A—C12A—C13A	1.4 (4)	C10B—C11B—C12B—C13B	−1.5 (4)
C14A—C11A—C12A—C13A	−176.8 (2)	C14B—C11B—C12B—C13B	177.9 (3)
C11A—C12A—C13A—C8A	−0.4 (4)	C11B—C12B—C13B—C8B	0.8 (4)
C11A—C12A—C13A—C15A	−180.0 (2)	C11B—C12B—C13B—C15B	−179.1 (3)
C9A—C8A—C13A—C12A	−0.8 (3)	C9B—C8B—C13B—C12B	1.0 (4)
C7A—C8A—C13A—C12A	179.2 (2)	C7B—C8B—C13B—C12B	−178.1 (2)
C9A—C8A—C13A—C15A	178.7 (2)	C9B—C8B—C13B—C15B	−179.0 (2)
C7A—C8A—C13A—C15A	−1.2 (4)	C7B—C8B—C13B—C15B	1.8 (4)
C12A—C11A—C14A—F1A	−151.1 (2)	C10B—C11B—C14B—F2B	−142.1 (3)
C10A—C11A—C14A—F1A	30.6 (4)	C12B—C11B—C14B—F2B	38.5 (4)
C12A—C11A—C14A—F3A	−30.4 (4)	C10B—C11B—C14B—F1B	−23.1 (4)
C10A—C11A—C14A—F3A	151.4 (3)	C12B—C11B—C14B—F1B	157.5 (3)
C12A—C11A—C14A—F2A	89.1 (3)	C10B—C11B—C14B—F3B	97.5 (4)
C10A—C11A—C14A—F2A	−89.1 (3)	C12B—C11B—C14B—F3B	−81.9 (4)
C12A—C13A—C15A—F4A	−120.3 (2)	C12B—C13B—C15B—F5B	0.8 (4)
C8A—C13A—C15A—F4A	60.1 (3)	C8B—C13B—C15B—F5B	−179.1 (2)
C12A—C13A—C15A—F5A	0.2 (3)	C12B—C13B—C15B—F4B	−120.0 (3)
C8A—C13A—C15A—F5A	−179.4 (2)	C8B—C13B—C15B—F4B	60.1 (3)
C12A—C13A—C15A—F6A	119.4 (2)	C12B—C13B—C15B—F6B	120.8 (3)
C8A—C13A—C15A—F6A	−60.2 (3)	C8B—C13B—C15B—F6B	−59.1 (3)

### Hydrogen-bond geometry (Å, °)

**Table:**

*D*—H···*A*	*D*—H	H···*A*	*D*···*A*	*D*—H···*A*
N2A—H2NA···O1B	0.86	2.05	2.856 (3)	156
N2B—H2NB···O1A^i^	0.86	2.10	2.908 (3)	155
C7A—H7A···O1B	0.93	2.23	3.055 (3)	147
C7B—H7B···O1A^i^	0.93	2.36	3.158 (3)	144
C2B—H2B···F1A^ii^	0.93	2.52	3.294 (3)	141
C9A—H9A···F2B^iii^	0.93	2.41	3.162 (4)	138
C12B—H12B···O1W^iv^	0.93	2.58	3.408 (5)	149
O1W—H1WA···F2B^iv^	0.84	2.01	2.845 (5)	180
O1W—H1WB···N1B^v^	0.84	2.09	2.932 (5)	180

Symmetry codes: (i) *x*−1, *y*, *z*; (ii) *x*−2, −*y*+3/2, *z*−1/2; (iii) −*x*+2, −*y*+2, −*z*+1; (iv) −*x*+1, −*y*+2, −*z*+1; (v) *x*+1, −*y*+3/2, *z*+1/2.
